# Book Reviews

**Published:** 1902-05

**Authors:** 


Diseases of the Intestines. Their Special Pathology, Diagnosis and Treatment. With sections on anatomy and physiology, microscopic and chemic examination of the intestinal contents, secretions, feces and urine; intestinal bacteria and parasites; surgery of the intestines; dietetics; diseases of the rectum, etc. By John C. Hemmeter, M. D., Ph. D., Professor in the Medical Department of the University of Maryland; Consultant to the University and Director to the Clinical Laboratory, etc. In two volumes. Volume I and II. With many original illustrations, some of which are in colors. Large 8 vos., pp. 742-680. Philadelphia: P. Blakiston's Son & Co. 1901-1902. [Price, $5.00 per volume.]

The first volume of this great work came from the press in the autumn of 1901; the second volume has but lately issued. They should be considered together in order to be appreciated, as they are consecutive to each other, the second being a continuation of the first, and when viewed in conjunction with the author's previous work on diseases of the stomach, help to constitute a complete treatise on diseases of the gastro-intestinal tract.

The first volume considers the anatomy and physiology of the intestinal tract; methods of diagnosis; therapy and materia medica of intestinal diseases; diarrhea, constipation, enteralgia, and enterodynia; meteorism, dystrypsia, enteritis, colitis, dysentery, intestinal ulcers, and intestinal neoplasms. These several sections are elaborated with much detail; giving many original suggestions relating to diagnosis, clinical research, and histological study.

In the second volume the medical side of appendicitis is fully set forth and then follow, in consecutive order, sections devoted to the consideration of tuberculosis, syphilis, and actinomycosis of the intestines; the occlusions, contusions, rupture, enterorrhagia and intestinal surgery, the borderline between medicine and surgery being set forth under the latter head; atrophy, abnormalities of form and position; thrombosis, embolism, amyloidosis; neuroses of the intestine; intestinal parasites, and, finally, diseases of the rectum are comprehensively dealt with, in a chapter prepared by Dr. Thomas C. Martin, of Cleveland. In the first volume is an important chapter upon the examination of the feces and urine, prepared by Dr. Harry Adler, of Baltimore.

The foregoing group of the general subjects that form the bulk of the treatise will convey but a slight idea of its real value or the comprehensiveness of its scope, still less of the minute scientific detail or great practical character of the work; but, perhaps, enough to afford the reader a hint as to its importance as a contribution to the literature of gastro-intestinal disease,—indeed, the first of strictly American origin that approaches anything like completeness, or that has claim to such distinction.

Dietetic treatment is becoming more and more important as it becomes better and better understood, and Hemmeter has made a careful study of the principles which are involved in the therapy of diet. He applies them with scientific skill, and makes dietary prophylaxis, so important in many diseases, not only fascinating as a study but practical and effective as a treatment.

The general physician will be greatly aided in the reading and consulting of this work and will gain a knowledge of dietary management not heretofore as effectively set forth or as clearly demonstrated. He will be convinced by its logic and become an exponent of the more modern and sensible view, that not alone is cure due to drug ingestion, but often to well regulated methods and habits of life, especially including a proper dietary regimen.

A SYSTEM OF PHYSIOLOGIC THERAPEUTICS. A Practical Exposition of the methods, other than Drug-giving, useful in the prevention of Disease and in the Treatment of the Sick. Edited by SOLOMON SOLIS COHEN, A. M., M. D., Professor of Medicine and Therapeutics in the Philadelphia Polyclinic; Lecturer on Clinical Medicine at Jefferson Medical College; Physician to the Philadelphia Hospital and to the Rush Hospital for Consumption, etc. In eleven 8vo volumes. American, English, German and French authors. Volume VI. Dietotherapy and Food in Health. By NATHAN S. DAVIS, Jr., A. M., M. D., Professor of the Principles and Practice of Medicine in Northwestern University Medical School; Physician to Mercy Hospital and Wesley Hospital, Chicago; Member American Medical Association, etc. Philadelphia: P. Blakiston's Son & Co., 1012 Walnut Street. 1901. [Price for the set complete, $27.50 net.]

The progress of this useful series of books is watched with interest. In it subjects are discussed that are too much neglected by the average physician. Specialists, in particular, are prone to overlook the care of the general system in their eagerness to correct the particular fault for which they may be consulted.

This number of the series is of particular importance because it deals with dietetics,—a subject that bears the closest relation to health and disease. It is one, moreover, that deeply concerns each individual, because its laws are violated daily by people of intelligence, who expect a doctor to prescribe a magical drug that will correct at once the errors of years.

The book before us is divided into two parts; the first considers the general principles of diet, and diet in health; the second part deals with diet in disease. To know how to properly feed the sick is only second to a knowledge of how to feed those who consider themselves well. In the first part, the several important articles of diet are described, their value set forth, and their dangers warned against. In the second part the diet applicable to the most common or important diseases is announced, and directions for its preparation and administration are given. It is a book that every intelligent person may read with profit.

THE AMERICAN YEAR-BOOK OF MEDICINE AND SURGERY FOR 1902. A Yearly Digest of Scientific Progress and Authoritative Opinion in all branches of Medicine and Surgery, drawn from journals, monographs and text-books of the leading American and foreign authors and investigators. Arranged, with critical editorial comments, by eminent American specialists, under the editorial charge of GEORGE M. GOULD, A.M , M.D. In two volumes—Volume I, including General Medicine, octavo, 700 pages, illustrated; Volume II, General Surgery, octavo, 684 pages, illustrated. Philadelphia and London: W. B. Saunders & Co. 1902. [Price per volume: Cloth, $3.00 net; half-morocco, $3.75 net.]

This work continues to be issued in two volumes, one on medicine and one on surgery,—a most excellent plan. It contains a most satisfactory selection of useful literature gleaned from the best periodicals, text-books, monographs and other sources, carefully arranged and scientically commented upon, by men competent to interpret their value and place.

The illustrations though not numerous are well chosen and the general make-up of the book is most satisfactory. It is a work that cannot be reviewed in the ordinary sense of the term, but it must be seen to be appreciated. It is very helpful to the over-worked physician.

